# Supplementary material for: Protocol for the first large-scale emergency care-based longitudinal cohort study of recovery after sexual assault: the Women’s Health Study
Source: BMJ Open. 2019 Nov 21;9(11):e031087. doi: 10.1136/bmjopen-2019-031087 (PMC6887008; doi:10.1136/bmjopen-2019-031087)
Supplement: Author's manuscript [file bmjopen-2019-031087.draft_revisions.pdf]

BMJ Open

BMJ Open is committed to open peer review. As part of this commitment we make the peer review history of every article we publish publicly available.

When an article is published we post the peer reviewers' comments and the authors' responses online. We also post the versions of the paper that were used during peer review. These are the versions that the peer review comments apply to.

The versions of the paper that follow are the versions that were submitted during the peer review process. They are not the versions of record or the final published versions. They should not be cited or distributed as the published version of this manuscript.

BMJ Open is an open access journal and the full, final, typeset and author-corrected version of record of the manuscript is available on our site with no access controls, subscription charges or pay-per-view fees (http://bmjopen.bmj.com).

If you have any questions on BMJ Open's open peer review process please email info.bmjopen@bmj.com

BMJ Open

Protocol for the first large-scale emergency care-based longitudinal cohort study of recovery after sexual assault: the Women's Health Study

Journal:	*BMJ Open*
Manuscript ID	bmjopen-2019-031087
Article Type:	Protocol
Date Submitted by the Author:	17-Apr-2019
Complete List of Authors:	Short, Nicole; Medical University of South Carolina, Psychiatry;<br>Sullivan, Jenyth; UNC-Chapel Hill<br>Soward, April; UNC-Chapel Hill<br>Bollen, Kenneth; University of North Carolina at Chapel Hill, Department of Psychology and Neuroscience and Department of Sociology<br>Liberzon, Israel; Texas A&M University System Health Science Center College of Medicine<br>Martin , Sandra; UNC-Chapel Hill<br>Rauch, S; Emory University<br>Bell, Kathy; Tulsa Forensic Nursing<br>Rossi, Catherine; Cone Health<br>Lechner, Megan; University Of Colorado Health Colorado Springs<br>Novak, Carissa; UNC-Chapel Hill<br>Witkemper, Kristen; UNC-Chapel Hill<br>Kessler, Ronald; Harvard Medical School<br>McLean, Samuel; UNC-Chapel Hill
Keywords:	posttraumatic stress, sexual assault, pain

SCHOLARONE™
Manuscripts

Adrian Aldcroft
Editor-in-Chief
BMJ Open

Dear Mr. Aldcroft and colleagues,

Please consider the enclosed study protocol for our ongoing NIH-funded prospective observational study of recovery after sexual assault for publication in *BMJ Open*. The study was funded by United States National Institutes of Health R01AR064700.

All authors contributed to the conception and/or design of this protocol. All authors have critically read this manuscript and have made their revisions, and have approved this manuscript for submission.

The authors of this protocol disclose that in the past 3 years, Dr. Kessler received support for his epidemiological studies from Sanofi Aventis; was a consultant for Johnson & Johnson Wellness and Prevention, Sage Pharmaceuticals, Shire, Takeda; and served on an advisory board for the Johnson & Johnson Services Inc. Lake Nona Life Project. Kessler is a co-owner of DataStat, Inc., a market research firm that carries out healthcare research. There are no other conflicts of interest to disclose. All authors have read and approved the manuscript. This manuscript has not been previously published nor is it under consideration for publication elsewhere.

Nicole Short will serve as the corresponding author during the manuscript submission process and I will serve as the formal corresponding author for the published manuscript.

Sincerely,

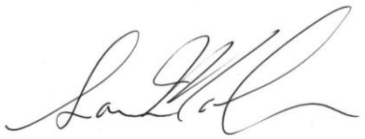

Samuel McLean, MD, MPH on behalf of the authors.

For peer review only

Protocol for the first large-scale emergency care-based longitudinal cohort study of recovery after sexual assault: the Women's Health Study

Nicole A. Short, Jenyth Sullivan, April Soward, Kenneth Bollen, Israel Liberzon, Sandra L. Martin, Sheila A.M. Rauch, Kathy Bell, Catherine Rossi, Megan Lechner, Carissa Novak, Kristen D. Witkemper, Ronald C. Kessler, Samuel A. McLean

Manuscript correspondence: Samuel A. McLean, MD, MPH
Institute for Trauma Recovery
University of North Carolina
211B W Cameron Ave
Chapel Hill, NC USA 27516

Phone: 919-843-5931

Fax: 919-966-7193

Email: Samuel_McLean@med.unc.edu

Running title: The Women's Health Study

Word Count: 3,512 excluding abstract (200), 1 tables, 0 figures, 67 references

Scientific Meeting Presentation: None

Funding: R01AR064700 funded by the following National Institutes of Health Institutes: NIAMS, NINDS, OD (ORWH), NINR, NIMH, and NICHD

For peer review only

ABSTRACT

Introduction: Worldwide, an estimated 10–27% of women are sexually assaulted during their lifetime. Despite the enormity of sexual assault as a public health problem, to our knowledge no large-scale prospective studies of experiences and recovery over time among women presenting for emergency care after sexual assault have been performed.

Methods and analysis: Women ≥18 years of age who present for emergency care within 72 hours of sexual assault to a network of treatment centers across the US are approached for study participation. Blood DNA and RNA samples and brief questionnaire and medical record data are obtained from women providing initial consent. Full consent is obtained at initial one week follow-up to analyze blood sample data and to perform assessments at one week, six weeks, six months, and one year. These assessments include evaluation of survivor life history, current health and recovery, and experiences with treatment providers, law enforcement, and the legal system.

Ethics and dissemination: This study is approved by the University of North Carolina at Chapel Hill's Institutional Review Board (IRB) and the IRB of each participating study site. We hope to present the results of this study to the scientific community at conferences and in peer-reviewed journals.

ARTICLE SUMMARY

Strengths and limitations of this study

- To our knowledge, this is the first large-scale prospective study of experiences and recovery among women presenting for emergency care after sexual assault.
- The study obtains detailed longitudinal data from the sexual assault survivors on adverse posttraumatic neuropsychiatric sequelae (APNS) over time after sexual assault, including posttraumatic stress, pain, somatic, cognitive, and depressive symptoms.
- The study collects both biologic data to gain new insights into the pathogenesis of APNS after sexual assault, and data to develop clinical prediction tools that identify those at high risk of APNS.
- The study obtains feedback from women sexual assault survivors regarding their experience with police, the legal system, and medical providers that can be used to improve services.
- Generalizability of study findings to women sexual assault survivors who do not report/come for care after sexual assault is not known.

For peer review only

INTRODUCTION

Worldwide, an estimated 10-27% of women are sexually assaulted during their lifetime.[1] In the United States (US), as many as 1.9 million women are sexually assaulted each year.[2,3] Survivors of sexual assault experience a heavy burden of adverse posttraumatic neuropsychiatric sequelae (APNS), including fear, anxiety, and posttraumatic stress.[4] In addition, as many as half of sexual assault survivors experience clinically significant depression,[5] one quarter report suicidal ideation, and 2-19% attempt suicide.[4] Preliminary evidence from small prospective cohorts also suggests that acute and chronic pain and somatic symptoms frequently develop after sexual assault.[6-8] Amidst this tremendous physical and mental health suffering, 13-49% of sexual assault survivors develop an alcohol use disorder and 28-61% develop addiction to other substances.[9,10]

In the US, emergency care for women sexual assault survivors is most commonly provided by a nurse with specific training and expertise, termed a Sexual Assault Nurse Examiner (SANE). The emergency care of sexual assault survivors provided by a SANE includes a thorough medical and forensic history, a detailed physical examination to document evidence of trauma, evidence collection, and risk stratification and preventive interventions to reduce pregnancy and/or sexually transmitted disease. Unfortunately, no risk stratification and preventive interventions are employed to reduce APNS. This is because risk stratification tools for APNS do not exist, and because the developmental biology of ANPS is poorly understood.

This article describes the study protocol for the first large-scale emergency care-based longitudinal cohort study of recovery after sexual assault: the WHS. The goals of the study are to help address the above barriers to intervention, gain a better understanding of survivor experiences with treatment providers, law enforcement, and legal and health care systems to inform improvement, and to determine the incidence and recovery trajectories of a range of APNS.

For peer review only

METHODS/DESIGN

Study Sites

The WHS is a prospective, multi-site observational study of women presenting for emergency SANE care following sexual assault. Women are recruited at emergency care centers across the United States that are part of the Better Tomorrow Network, including: Albuquerque SANE Collaborative, Albuquerque, New Mexico; Tulsa Forensic Nurse Examiners, Tulsa, Oklahoma; Christiana Care SANE Program, Newark, Delaware; Austin SAFE, Austin, Texas; Hennepin HART, Minneapolis, Minnesota; Crisis Center of Birmingham, Birmingham, Alabama; Philadelphia SARC/Einstein Healthcare, Philadelphia, Pennsylvania; Denver Health SANE, Denver, Colorado; Wayne County SAFE, Detroit, Michigan; University of Louisville SAFE, Louisville, Kentucky; Memorial Health SANE, Colorado Springs, Colorado; DC SANE, Washington, DC; and Cone Health, Greensboro, North Carolina. Each site's IRB approved the study protocol. The Better Tomorrow Network data coordinating center is located at the University of North Carolina (UNC) at Chapel Hill North Carolina, USA, and all procedures were approved by the UNC IRB (13 – 3193).

Patient Inclusion Criteria

Women at least 18 years of age who present to a participating site for emergency care within 72 hours of assault are assessed for potential eligibility. Exclusion criteria include: inability to provide informed consent (e.g., due to intoxication, injury), pregnancy, living with one's assailant, presence of fracture, injury requiring hospital admission, inability to speak or read English, no telephone access, no mailing address, unwilling to provide a blood sample, incarceration, and other situations resulting in inability to follow the study protocols. Enrollment began in June 2015, study follow-ups are scheduled to be completed in 2020, with a target enrollment of 700 women.

Patient Screening and Initial Assessment

Treatment providers at study network sites notify on-call research associates (RAs) when a potentially eligible individual presents for emergency care after sexual assault. RAs approach potential participants at a time determined by the sexual assault survivor care provider(s). RAs provide a brief description of the study and request written consent to (1) perform a brief survey, (2) contact the survivor by telephone in 48-72 hours to explain the full study and ask if the individual is interested in participating, (3) access medical record/forensic examination information related to the sexual assault, and (4) collect blood samples (discarded if the participant does not subsequently consent to the full study at one week). Individuals are specifically informed that they are not being asked to decide about full study participation at the time of their emergency care. Participants are compensated $20 for completing the initial assessment.

RAs communicate with potential participants regarding consent for the initial evaluation either in-person or via the use of teleconsent. To obtain teleconsent, the RA introduces her/himself to the participant via live two-way video communication over tablet or laptop, and describes her/his role as a research associate in an ongoing study to learn more about the recovery process after trauma. Just as in-person, if the potential participant is willing, the RA reviews the study consent and requirements for participation and uses the online system to screen the potential participant for study eligibility. If eligible, the RA opens a copy of the initial consent on the tablet or laptop, and obtains an e-signature. The teleconsent platform complies with Health Insurance Portability and Accountability Act (HIPAA) requirements by (1) encrypting all transmitted data, (2) not storing patient information, and (3) keeping an audit trail of the consent process.[11] This teleconsent is used as a consent option because it has been shown to be a safe and valid method of obtaining consent in medical settings[11-18] and because it addresses barriers that prevent critically needed research in settings where it is difficult to consistently provide trained research staff. Teleconsent also addresses inequities created by the fact that research staff are more difficult to hire/provide in socioeconomically disadvantaged and/or rural areas.

Assessment at One Week

For peer review only

Participants who are reached by phone and express interest in study participation are scheduled for a one-week in-person assessment. Assessments take place either at the initial emergency care site or at a public community location acceptable to both the RA and study participant. At the beginning of the study visit, the RA obtains written informed consent to participate in follow-up evaluations one week, six weeks, six months, and one year after the assault and consent to utilize the blood samples obtained at the time of emergency care. After written informed consent is obtained, the one week evaluation is typically performed via a web-based self-report computer survey completed on a laptop computer. Use of a computerized self-report survey standardizes the assessment experience across RAs and sites, increases reporting of experiences that may be considered stigmatizing,[19] and facilitates honest feedback regarding the experience of being in the study. Computer self-report surveys have been shown to be a valid and acceptable means of obtaining research assessment data, including in socioeconomically disadvantaged populations recruited in emergency care settings.[20-22] The beginning of each web-based assessment includes introductory practice questions using the different questionnaire formats. After the completion of these practice questions, the RA moves to another location in the room, so that the study participant has privacy when recording her computer questionnaire responses. Participants are compensated $50 for completing the one-week assessment.

Assessment at Six Weeks, Six Months, and Twelve Months

Follow-up survey assessments at six weeks, six months, and twelve months may be completed in-person, or if the participant prefers, via logging into a secure website on a computer of their choice, or over the phone. The format of the web-based computer self-report surveys completed online are identical to the web-based computer self-report surveys completed by participants who elect to complete surveys in-person. Each online assessment contains specific completion instructions. Participants who complete follow-up assessments online independently may call the toll-free study phone number if they have any questions while completing the assessment. Participants are encouraged to complete these follow-up assessments either in-person or online, to maximize standardization. However, if participants are unable to complete these follow-up assessments either in-person or online, they may also complete them via telephone interview. Participants are compensated $50 for completing each follow-up assessment.

Contact Information Updates at Three Months and Nine Months

Participants are contacted three months and nine months after enrollment, and are asked to update their contact information. Participants are compensated $10 for each follow-up request.

Patient and Public Involvement

The study protocol was designed in collaboration with sexual assault nurse examiners; this team included sexual assault survivors. The study was reviewed and funded by the National Institutes of Health, which considers public funding priorities and maximizing the public good in scientific review and funding decisions. As described below, study participants provide feedback on burden and experiences in the research study at the end of each assessment. Study results are disseminated to the public via presentations, publications, and media coverage, and via provision of study results to individual sexual assault treatment facilities and to state coalitions against sexual assault in states in which participating care sites are located.

Study Assessments (Table 1)

Assessments and data collection at the time of emergency care

Pain severity (0-10 numeric rating scale score) is assessed in each of 23 body regions using an adapted version of the Regional Pain Scale (RPS).[23] This scale has been shown to be a valid, reliable measure of pain location and distribution.[23] Pain severity in the head and face, breast area, pelvic/genital region, hands, and feet are also assessed. Blood DNA and RNA are collected using PAXgene DNA (8.5cc) and RNA (2.5cc) storage tubes, respectively. Samples are frozen at the study site and are shipped in batches to the University of North Carolina. A barcode-based study tracking system tracks each tube throughout the storage and shipment process to maintain chain of custody.

For peer review only - http://bmjopen.bmj.com/site/about/guidelines.xhtml

Medical records regarding emergency care after sexual assault are password protected and then uploaded to the encrypted, secure study database by the site RA. If some of the participant's emergency care was provided at a treatment location separate from the location of the SANE exam, these records are also obtained by study staff. Medical record data are abstracted by RAs using detailed protocols and variable definitions. Quality assurance and control of data abstraction are assessed via double data entry and discrepancies are adjudicated by the study PI.

Assessments at One Week, Six Weeks, Six Months, and One Year

Assessments at one week, six weeks, six months, and one year include the following:

General physical and mental health status: The Patient-Reported Outcomes Measurement Information System (PROMIS) Global Health assessment is used to assess general pre-assault health at the one-week follow-up. At subsequent time points, this measure is used to assess health status.[24]

Somatic symptoms: Pre-injury somatic symptoms during the week prior to the sexual assault are assessed at one-week follow-up using a 21-question symptom inventory. Somatic symptoms during the past week are additionally assessed at each follow-up time point.

Posttraumatic stress disorder symptoms, past trauma, and resilience: Lifetime trauma and PTSD history are assessed at one week using questions from an adapted version of the Life Events Checklist.[25] Lifetime PTSD symptoms related to prior trauma are assessed with an abbreviated and adapted version of the PCL-5[26,27] that explicitly instructs the respondent to focus only on symptoms related to prior stressors, not the recent sexual assault. An adapted version of the Trait Resilience scale of Kessler, et al [28-30] is administered at one week and the Adverse Childhood Experiences (ACE [31,32]) Score Calculator is administered at six weeks.[32] Recent sexual assault-related PTSD symptoms are assessed using the PCL-5[27,33] with the addition of the two DSM-IV-related items from the PCL-C[26,27,34].

Depressive and other anxiety-related assessments symptoms: Short-form versions of the PROMIS Depression and Anxiety assessments[24] (Short Form 8b and Short Form 8a, respectively) are used at the one-week follow-up to assess depressive and anxiety symptom burden in the week prior to assault. These assessments are also used at the six-week, six-month, and twelve-month follow-ups to assess symptoms during the past week. In addition, a brief version of the Anxiety Sensitivity Index - 3 (ASI-3) is administered at one week follow-up.[35,36]

Pain symptoms: The same assessment method used to evaluate the severity and location of pain at the time of SANE exam are used to evaluate both reported pre-assault pain and post-assault pain symptoms. Pain symptoms assessed at each time point are specifically termed "pain/tenderness" and "pain or aching", as past experience with sexual assault survivors indicates that some survivors otherwise interpret questions about "pain" to include "emotional pain." At the one-week assessment, after being asked about the location and severity of any pain symptoms since the assault, participants are also asked about the location and severity of any pain symptoms during the week before assault. If participants report pain in a body region at six-week, six-month, or twelve-month follow-up, after rating the severity of pain in that region participants are asked to indicate if this pain is related to the sexual assault. In addition to pain severity assessments in each body region, at each follow-up timepoint overall pain is also assessed using a 0-10 NRS and pain interference during the past week is assessed using questions from the Brief Pain Inventory.[37]

Substance use: Drug, alcohol, and tobacco use during the month prior to the month of assault are assessed at the one-week follow-up using an adapted version of the CIDI-SC assessment of substance use disorder of Kessler, et al.[29,30] This time point was chosen to exclude the timeframe of the assault. At all subsequent follow-ups, use during the past month is assessed.

Demographic information: Information including age, race/ethnicity, education level, income level, marital status, work status, and height and weight are obtained from questionnaire items and the medical record.

Past medical history: Participant past medical history is obtained from the medical records.

Medication use: Participant medication use is assessed at each follow-up evaluation.

Experiences of sexual assault survivors with care providers and the health care and legal systems: Sexual assault survivor experiences, including experiences with the SANE program, legal system, and police, as well as health services utilization and injuries since assault, are assessed using standardized questionnaires.

Sexual assault survivor experiences with the study in general and with individual follow-up assessments: After each interview, participants complete an adapted version of the Reactions to Research Participation Questionnaire (RRPQ)[38,39] and open-ended questions about their experiences as a study participant and sexual assault survivor.

Analyses

Study results will be shared with the scientific community via conferences and peer-reviewed journal articles. Planned analyses fall into four broad areas.

1. Assess the incidence over time of a range of APNS, using both traditional and experimental/state-of-the-art classification systems. No large-scale emergency care-based multi-site studies of sexual assault survivors have been conducted, thus the incidence of the full spectrum of APNS over time after sexual assault remains poorly understood. This study will assess a range of APNS, as well as multivariate patterns of comorbidity among these outcomes and the impact of these outcomes on general physical and mental health over time. APNS will be assessed using both traditional classifications (e.g., posttraumatic stress disorder, depression, pain, and post-concussion symptoms), and also more discrete, homogenous outcomes (e.g., avoidance, re-experiencing, numbing, hopelessness, etc.). These homogenous outcomes will, to the extent possible, build upon the National Institute of Mental Health Research Domain Criteria (RDoC) classification system (https://bit.ly/2pudCZH). Subsequent analyses will attempt to group sexual assault survivors according to the combinations of these discrete outcomes as they develop. Classification systems such as RDoC attempt to overcome two important limitations to traditional classifications: (1) they are not indexed to specific biological processes or components of brain functioning, which often evolved based

2. Gain new understanding of the pathogenesis of adverse posttraumatic neuropsychiatric outcomes after sexual assault. As noted above, a major barrier to developing more effective preventive interventions is that the pathogenesis of APNS after sexual assault remains poorly understood. An evaluation of risk factors and developmental processes involved in the temporal unfolding of adverse posttraumatic neuropsychiatric outcomes after sexual assault will be evaluated using both traditional and molecular epidemiologic methods, with complementary cell culture and animal mechanistic studies where valuable. Available data indicate that biologic samples collected in the immediate aftermath of trauma can provide valuable insights into the pathogenesis of APNS (e.g.,[40-50]). The goal of studies performed with these data (e.g., genetic, epigenetic, gene expression, miRNA studies) will be to gain improved understanding to inform the development of more effective preventive/recovery interventions for sexual assault survivors.

3. Derive risk prediction tools that identify sexual assault survivors at high risk of specific adverse posttraumatic outcome(s). For more than 50 years, women sexual assault survivors presenting for emergency care after assault have been risk stratified for pregnancy and sexually transmitted disease, and those at high risk have received preventive interventions. However, such risk stratification tools exist to help identify women at high risk of APNS.[4] Survivor assault characteristics, demographic characteristics, life history, pre-assault health status, acute posttraumatic symptoms, and biologic characteristics will be used in an effort to develop clinical decision support tools that identify survivors at high risk of one or more APNS. These clinical decision support tools will be developed using machine learning (ML) methods,[51] training and validation samples, and internal cross-validation of training samples to minimize over-fitting. A number of different machine learning algorithms will be examined, such as naïve Bayes, ensemble regression trees (random forests), penalized regression (elastic net), algorithms that allow for nonlinearities and non-additivities in stepwise trees with embedded splines (gradient boosting), and support vector machines. The value of combining results across these different algorithms using the super learner ensemble method will also be assessed.[52-56] Tiering and targeting will be used to try to limit the number of items needed in prediction tools. Tiering refers to nested ML analyses based on successively adding more costly predictors to the models, where cost is defined in terms of both time (e.g., number of questions that SANE needs to ask to complete) and processing (e.g., costs of genetic testing, neuroimaging, etc.). Targeting refers to determining subsets of patients that vary in the extent to which prediction accuracy over a clinical decision threshold varies depending on a given level of

For peer review only - http://bmjopen.bmj.com/site/about/guidelines.xhtml

tiering. For example, screening tests are often used to determine whether individual patients need more complex and expensive tests. The equivalent in our context will be to determine the cross-validated predicted values based on initial predictors that indicate the need for further data.

4. Obtain feedback from sexual assault survivors regarding their experiences with health care providers, law enforcement, and the legal system, and their experiences with the present research study. The lack of large-scale research networks for sexual assault survivors has hampered the ability to obtain feedback from survivors regarding services they receive. Such feedback is critical for continued quality improvement and to ensure that health, law enforcement, and legal systems designed for sexual assault survivors are indeed "survivor-centered." Similarly, it is critical for research studies to ensure that their protocols are not viewed as negative experiences by sexual assault survivor cohorts taking part in them. Analyses will be performed which evaluate survivor experiences with these services and with the research protocol.

Ethics and Dissemination

Although the safety of participants is of paramount importance to any research study, this is a matter of particular concern in studies of traumatized individuals. Study participants are told that they may choose not to answer any questions that cause discomfort and that they will be paid the full financial incentive regardless of whether or not they decide to skip self-report questionnaire items or stop the questionnaire. As noted above, study participants who choose to participate at the time of the emergency care provide written or electronic informed consent only for the initial evaluation. At the beginning of the one-week assessment, in-person written consent is obtained to perform the one-week assessment and follow-up assessments. The research assessments never include details of/questions regarding the assault experience itself. This information is obtained from emergency care records. During enrollment in the study, participants are informed that they will be contacted to learn more about the recovery process after trauma. The study is framed in terms of "recovery," as we believe that it is very important to help create an expectation of healing in study participants since previous research suggests that participant expectation may potentially influence long-term outcomes after trauma.[57] One-week assessments are performed via telephone or self-report computer-based questionnaire. Subsequent surveys at six weeks, six months, and one year are also offered either via telephone or self-report computer-based questionnaire. This increases privacy and anonymity and encourages candid participant feedback regarding study participation. Assessment modalities (i.e.., in-person, online, telephone) are offered depending on participants' preference. This methodology balances our goals of providing as much anonymity as possible while allowing those participants the opportunity to continue to with the study who have travel issues or do not have internet access at a follow-up time point.

Study participants receive the exact same survivor services and follow-up referrals/care as other survivors presenting for care. All survivors are actively referred for mental and physical health services through usual post-assault care follow-up services managed by their emergency care providers and are encouraged to utilize a rape crisis advocate to help them receive this care. Participants who report substantial distress and/or who appear to be developing significant anxiety and/or depressive symptoms are encouraged to utilize local mental health services. In addition, study participants are provided with a book (free of charge), *Recovering from Rape*[58] or *Life, Reinvented*,[59] which is written specifically to help women in the early aftermath of sexual assault.

Concluding Summary

The WHS is a unique large-scale emergency care-based longitudinal cohort study of recovery in the 12 months after sexual assault. The study seeks to increase understanding of the incidence, pathogenesis, and trajectories of adverse neuropsychiatric sequelae, to develop clinical decision support tools that effectively predict such outcomes, and to obtain feedback from survivors regarding the services they receive. Study success is based on our study participants, who share their experiences with the understanding that researchers will use this information to try to improve the care of survivors in the future. We are grateful for their efforts and recognize our responsibility.

For peer review only

FUNDING

This work was supported by NIH R01 AR064700. The content is solely the responsibility of the authors and does not necessarily represent the official views of the NIH.

COMPETING INTERESTS

In the past 3 years, Dr. Kessler received support for his epidemiological studies from Sanofi Aventis; was a consultant for Johnson & Johnson Wellness and Prevention, Sage Pharmaceuticals, Shire, Takeda; and served on an advisory board for the Johnson & Johnson Services Inc. Lake Nona Life Project. Kessler is a co-owner of DataStat, Inc., a market research firm that carries out healthcare research.

CONTRIBUTORS

Samuel McLean conceived of the study. Nicole Short, Jenyth Sullivan, April Soward, Kenneth Bollen, Israel Liberzon, Sandra L. Martin, Sheila A.M. Rauch, Kathy Bell, Catherine Rossi, Megan Lechner, Ronald C. Kessler, Carissa Novak, Kristen Witkemper, and Samuel McLean helped to design the final study protocol. All authors reviewed the manuscript.

For peer review only

Table 1
Study question domains and self-report measures administered at each time point

Domain	Measure	Initial	1-Wk	6-Wk	6-Mo	12-Mo
Assault Characteristics	Data extraction from medical record	X				
Acute Pain	Pain Severity Numeric Rating Scale[60]	X	X	X	X	X
Pain Related to the Assault	Pain Severity Numeric Rating Scale[60]		X	X	X	X
Pain Interference	Brief Pain Inventory[61]		X	X	X	X
Pre-Assault Pain	Pain Severity Numeric Rating Scale[60]		X			
Current Medication Use	Standard Items		X	X	X	X
Survivor Experience	SANE Care, advocate, post-exposure prophylaxis		X			
	Experience with police, post-exposure prophylaxis, health-services utilization, out-of-pocket expenses			X		
	Experience with police, health services utilization, out-of-pocket expenses				X	
	Experience with police, experience with legal system, health services utilization, out-of-pocket expenses					X
Somatic Symptoms	Numeric rating scale score of 21 common symptoms		X	X	X	X
Pre-Assault Somatic Symptoms	Standard Items		X			
Posttraumatic Stress Symptoms	PTSD Checklist – Situation (PCL-S)[26,27]		X	X	X	X
Pre-Assault Trauma Exposure	Adapted version of the Life Events Checklist[25]		X			
Pre-Assault Posttraumatic Stress	Adapted version of PCL-5[26,27]		X			
Childhood Trauma Exposure	Childhood Adversity Exposures (ACE)[31]			X		
Resilience	Trait Resiliency[28]		X			
General Health	PROMIS Global Health – Physical Component[24]		X	X	X	X
New Health Status	Standard items			X	X	X

For peer review only

Depression	PROMIS – Short Form 8b[24]	X	X	X	X
Anxiety	PROMIS – Short Form 8a[24]	X	X	X	X
Substance Use	Adapted version of Composite International Diagnostic Interview Screening Scales (CIDI-SC)[29]	X	X	X	X
Demographics	Standard items	X			
Research Experience	Reactions to Research Participation Questionnaire Revised[39]	X	X	X	X

Note: SANE = sexual assault nurse examiner; PTSD = posttraumatic stress disorder; PROMIS = Patient-reported Outcomes Measurement Information System; CIDI-SC = Composite International Diagnostic Interview – Screening Scale.

For peer review only

REFERENCES

1. García-Moreno C, World Health Organization. Dept. of Gender and Women's Health. WHO multi-country study on women's health and domestic violence against women : initial results on prevalence, health outcomes and women's responses. Geneva: World Health Organization; 2005.
2. Breiding MJ, Smith SG, Basile KC, Walters ML, Chen J, Merrick MT. Prevalence and characteristics of sexual violence, stalking, and intimate partner violence victimization—National Intimate Partner and Sexual Violence Survey, United States, 2011. American journal of public health 2015;105:E11.
3. Peterson C, DeGue S, Florence C, Lokey CN. Lifetime economic burden of rape among US adults. American journal of preventive medicine 2017;52:691-701.
4. Campbell R, Dworkin E, Cabral G. An ecological model of the impact of sexual assault on women's mental health. Trauma, Violence, & Abuse 2009;10:225-46.
5. Acierno R, Brady K, Gray M, Kilpatrick DG, Resnick H, Best CL. Psychopathology following interpersonal violence: A comparison of risk factors in older and younger adults. Journal of Clinical Geropsychology 2002;8:13-23.
6. Ballina LE, Ulirsch JC, Soward AC, et al. mu-Opioid Receptor Gene A118G Polymorphism Predicts Pain Recovery After Sexual Assault. J Pain 2012.
7. McLean SA, Soward AC, Ballina LE, et al. Acute severe pain is a common consequence of sexual assault. J Pain 2012;13:736-41.
8. Ulirsch JC, Ballina LE, Soward AC, et al. Pain and somatic symptoms are sequelae of sexual assault: results of a prospective longitudinal study. Eur J Pain 2014;18:559-66.
9. Ullman SE, Filipas HH, Townsend SM, Starzynski LL. Psychosocial correlates of PTSD symptom severity in sexual assault survivors. Journal of traumatic stress 2007;20:821-31.
10. Ullman SE, Filipas HH, Townsend SM, Starzynski LL. Correlates of comorbid PTSD and drinking problems among sexual assault survivors. Addictive behaviors 2006;31:128-32.
11. Welch B, Marshall E, Qanungo S, et al. Teleconsent: A novel approach to obtain informed consent for research. Contemporary Clinical Trials Communications 2016;3:74-9.
12. Schenker Y, Meisel A. Informed consent in clinical care: practical considerations in the effort to achieve ethical goals. JAMA 2011;305:1130-1.
13. Bobb MR, Van Heukelom PG, Faine BA, et al. Telemedicine provides noninferior research informed consent for remote study enrollment: a randomized controlled trial. Academic Emergency Medicine 2016;23:759-65.
14. Sonne SC, Andrews JO, Gentilin SM, et al. Development and pilot testing of a video-assisted informed consent process. Contemporary clinical trials 2013;36:25-31.
15. Rojas SV, Gagnon M-P. A systematic review of the key indicators for assessing telehomecare cost-effectiveness. Telemedicine and e-Health 2008;14:896-904.
16. Witherspoon E. UNC's CTSA hosts experts in telemedicine and health information technology. News Room-UNC Healthcare Vital Signs 2010.

For peer review only

17. Cone Health. Health Online: How the Internet is Revolutionizing Patient Service. 2017.
18. Narum E. Making the Grade: School-Based Telemedicine and Parental Consent. Making the Grade. San Diego Law Review 2016;53:745-51.
19. Turner CF, Ku L, Rogers SM, Lindberg LD, Pleck JH, Sonenstein FL. Adolescent sexual behavior, drug use, and violence: increased reporting with computer survey technology. Science (New York, NY) 1998;280:867-73.
20. Gregor MA, Shope JT, Blow FC, Maio RF, Weber JE, Nypaver MM. Feasibility of using an interactive laptop program in the emergency department to prevent alcohol misuse among adolescents. Annals of Emergency Medicine 2003;42:276-84.
21. McLean SA, Blow FC, Walton MA, et al. Rates of At‐risk Drinking among Patients Presenting to the Emergency Department with Occupational and Nonoccupational Injury. Academic Emergency Medicine 2003;10:1354-61.
22. Rhodes KV, Lauderdale DS, Stocking CB, Howes DS, Roizen MF, Levinson W. Better health while you wait: a controlled trial of a computer-based intervention for screening and health promotion in the emergency department. Annals of Emergency Medicine 2001;37:284-91.
23. Wolfe F. Pain extent and diagnosis: development and validation of the regional pain scale in 12,799 patients with rheumatic disease. The Journal of rheumatology 2003;30:369-78.
24. Cella D, Riley W, Stone A, et al. The Patient-Reported Outcomes Measurement Information System (PROMIS) developed and tested its first wave of adult self-reported health outcome item banks: 2005–2008. Journal of clinical epidemiology 2010;63:1179-94.
25. Gray MJ, Litz BT, Hsu JL, Lombardo TW. Psychometric properties of the life events checklist. Assessment 2004;11:330-41.
26. Weathers F, Litz B, Herman D, Huska J, Keane T. The PTSD checklist (PCL): Reliability, validity, and diagnostic utility. Annual Convention of the International Society for Traumatic Stress Studies. San Antonio, TX1993.
27. Weathers FW, Litz BT, Keane TM, Palmieri PA, Marx BP, Schnurr PP. The PTSD checklist for DSM-5 (PCL-5). National Center for PTSD; 2013.
28. Campbell‐Sills L, Kessler RC, Ursano RJ, et al. Predictive validity and correlates of self‐assessed resilience among US Army soldiers. Depression and anxiety 2018;35:122-31.
29. Kessler RC, Santiago PN, Colpe LJ, et al. Clinical reappraisal of the Composite International Diagnostic Interview Screening Scales (CIDI‐SC) in the Army Study to Assess Risk and Resilience in Servicemembers (Army STARRS). International journal of methods in psychiatric research 2013;22:303-21.
30. Kessler RC, Calabrese JR, Farley P, et al. Composite International Diagnostic Interview screening scales for DSM-IV anxiety and mood disorders: Cambridge Univ Press; 2013.
31. Felitti VJ, Anda RF, Nordenberg D, et al. Relationship of childhood abuse and household dysfunction to many of the leading causes of death in adults: The Adverse Childhood Experiences (ACE) Study. American journal of preventive medicine 1998;14:245-58.

For peer review only

32. Butchart A, Harvey AP, Mian M, Furniss T. Preventing child maltreatment: a guide to taking action and generating evidence. 2006.
33. Blanchard EB, Jones-Alexander J, Buckley TC, Forneris CA. Psychometric properties of the PTSD Checklist (PCL). Behaviour research and therapy 1996;34:669-73.
34. Weathers FW, Huska JA, Keane TM. PCL-C for DSM-IV. Boston: National Center for PTSD-Behavioral Science Division 1991.
35. Reiss S, Peterson RA, Gursky DM, McNally RJ. Anxiety sensitivity, anxiety frequency and the prediction of fearfulness. Behavior Research and Therapy 1986;24:1-8.
36. Taylor S, Zvolensky MJ, Cox BJ, et al. Robust dimensions of anxiety sensitivity: development and initial validation of the Anxiety Sensitivity Index-3. Psychol Assess 2007;19:176-88.
37. McCaffrey M, Pasero C. Pain: Clinical Manual. St. Louis, MO: Mosby, Inc.; 1999.
38. Griffin M, Resick P, Waldrop A, Mechanic M. Participation in trauma research: is there evidence of harm? J Trauma Stress 2003;16:221.
39. Newman E, Kaloupek D. Reactions to research participation questionnaire. Available from E Newman, Department of Psychology, University of Tulsa 1996;600:74114-3189.
40. Linnstaedt SD, Walker MG, Parker JS, et al. microRNA circulating in the early aftermath of motor vehicle collision predict persistent pain development and suggest a role for microRNA in sex-specific pain differences. Molecular Pain 2015;In Press.
41. Linnstaedt SD, Riker KD, Rueckeis CA, et al. A Functional riboSNitch in the 3' Untranslated Region of FKBP5 Alters MicroRNA-320a Binding Efficiency and Mediates Vulnerability to Chronic Post-Traumatic Pain. J Neurosci 2018;38:8407-20.
42. Linnstaedt SD, Hu J, Bortsov AV, et al. mu-Opioid Receptor Gene A118 G Variants and Persistent Pain Symptoms Among Men and Women Experiencing Motor Vehicle Collision. J Pain 2015;16:637-44.
43. Linnstaedt SD, Bortsov AV, Soward AC, et al. CRHBP polymorphisms predict chronic pain development following motor vehicle collision. Pain 2016;157:273-9.
44. Ballina LE, Ulirsch JC, Soward AC, et al. mu-Opioid receptor gene A118G polymorphism predicts pain recovery after sexual assault. J Pain 2013;14:165-71.
45. Ulirsch JC, Weaver MA, Bortsov AV, et al. No man is an island: living in a disadvantaged neighborhood influences chronic pain development after motor vehicle collision. Pain 2014;155:2116-23.
46. Orrey DC, Bortsov AV, Hoskins JM, et al. Catechol-O-methyltransferase genotype predicts pain severity in hospitalized burn patients. J Burn Care Res 2012;33:518-23.
47. Bortsov AV, Smith JE, Diatchenko L, et al. Polymorphisms in the glucocorticoid receptor co-chaperone FKBP5 predict persistent musculoskeletal pain after traumatic stress exposure. Pain 2013;154:1419-26.
48. Bortsov AV, Diatchenko L, McLean SA. Complex multilocus effects of catechol-O-methyltransferase haplotypes predict pain and pain interference 6 weeks after motor vehicle collision. Neuromolecular Med 2014;16:83-93.

For peer review only

49. Bortsov A, Diatchenko L, Swor RA, et al. COMT haplotypes predict pain intensity and interference 6 weeks after motor vehicle collision. Annual Meeting of the American Pain Society. New Orleans, LA2013.
50. Auvergne L, Bortsov AV, Ulirsch JC, et al. Association of Epidemiologic Factors and Genetic Variants Influencing Hypothalamic-Pituitary-Adrenocortical Axis Function With Postconcussive Symptoms After Minor Motor Vehicle Collision. Psychosom Med 2016;78:68-78.
51. Hastie T, Tibshirani R, Friedman JH. The elements of statistical learning : data mining, inference, and prediction. New York: Springer; 2009.
52. Kessler RC, Rose S, Koenen KC. How well can post‐traumatic stress disorder be predicted from pre‐trauma risk factors? An exploratory study in the WHO. World Mental Health Surveys World Psychiatry;2014:265‐74.
53. Kessler RC, Warner CH, Ivany C. Predicting suicides after psychiatric hospitalization in US Army soldiers: the army study to assess risk and resilience in service members (Army STARRS). JAMA psychiatry;2015:49–57.
54. van der Laan MJ PE, Hubbard AE. Super learner. Stat Appl Genet Mol Biol 2007:25.
55. Kessler R, van Loo H, Wardenaar K. Testing a machine-learning algorithm to predict the persistence and severity of major depressive disorder from baseline self-reports. Molecular Psychiatry in press.
56. Koenen KC, Kessler RC, Shalev AY. RO1:Identifying risk factors for ptsd by pooled analysis of current prospective studies. NIH:2014–9.
57. Mittenberg W, DiGiulio D, Perrin S, Bass A. Symptoms following mild head injury: expectation as aetiology. J Neurol Neurosur Ps 1992;55:200.
58. Ledray LE. Recovering from rape: Macmillan; 1994.
59. E. C. Life, Reinvented: Quantum Publishing Group; 2014.
60. Bijur PE, Latimer CT, Gallagher EJ. Validation of a verbally administered numerical rating scale of acute pain for use in the emergency department. Academic Emergency Medicine 2003;10:390-2.
61. Keller S, Bann CM, Dodd SL, Schein J, Mendoza TR, Cleeland CS. Validity of the brief pain inventory for use in documenting the outcomes of patients with noncancer pain. The Clinical journal of pain 2004;20:309-18.

For peer review only

BMJ Open

Protocol for the first large-scale emergency care-based longitudinal cohort study of recovery after sexual assault: the Women's Health Study

Journal:	*BMJ Open*
Manuscript ID	bmjopen-2019-031087.R1
Article Type:	Protocol
Date Submitted by the Author:	30-Aug-2019

Complete List of Authors:	Short, Nicole; Medical University of South Carolina, Psychiatry;<br>Sullivan, Jenyth; UNC-Chapel Hill<br>Soward, April; UNC-Chapel Hill<br>Bollen, Kenneth; University of North Carolina at Chapel Hill, Department of Psychology and Neuroscience and Department of Sociology<br>Liberzon, Israel; Texas A&M University System Health Science Center College of Medicine<br>Martin , Sandra; UNC-Chapel Hill<br>Rauch, S; Emory University<br>Bell, Kathy; Tulsa Forensic Nursing<br>Rossi, Catherine; Cone Health<br>Lechner, Megan; University Of Colorado Health Colorado Springs<br>Novak, Carissa; UNC-Chapel Hill<br>Witkemper, Kristen; UNC-Chapel Hill<br>Kessler, Ronald; Harvard Medical School<br>McLean, Samuel; UNC-Chapel Hill

**Primary Subject Heading**:	Sexual health
Secondary Subject Heading:	Emergency medicine
Keywords:	posttraumatic stress, sexual assault, pain

SCHOLARONE™ Manuscripts

Protocol for the first large-scale emergency care-based longitudinal cohort study of recovery after sexual assault: the Women's Health Study

Nicole A. Short, Jenyth Sullivan, April Soward, Kenneth Bollen, Israel Liberzon, Sandra L. Martin, Sheila A.M. Rauch, Kathy Bell, Catherine Rossi, Megan Lechner, Carissa Novak, Kristen D. Witkemper, Ronald C. Kessler, Samuel A. McLean

Manuscript correspondence: Samuel A. McLean, MD, MPH
Institute for Trauma Recovery
University of North Carolina
211B W Cameron Ave
Chapel Hill, NC USA 27516

Phone: 919-843-5931

Fax: 919-966-7193

Email: Samuel_McLean@med.unc.edu

Running title: The Women's Health Study

Word Count: 3,512 excluding abstract (200), 1 tables, 0 figures, 67 references

Scientific Meeting Presentation: None

Funding: R01AR064700 funded by the following National Institutes of Health Institutes: NIAMS, NINDS, OD (ORWH), NINR, NIMH, and NICHD

For peer review only

ABSTRACT

Introduction: Worldwide, an estimated 10–27% of women are sexually assaulted during their lifetime. Despite the enormity of sexual assault as a public health problem, to our knowledge no large-scale prospective studies of experiences and recovery over time among women presenting for emergency care after sexual assault have been performed.

Methods and analysis: Women ≥18 years of age who present for emergency care within 72 hours of sexual assault to a network of treatment centers across the US are approached for study participation. Blood DNA and RNA samples and brief questionnaire and medical record data are obtained from women providing initial consent. Full consent is obtained at initial one week follow-up to analyze blood sample data and to perform assessments at one week, six weeks, six months, and one year. These assessments include evaluation of survivor life history, current health and recovery, and experiences with treatment providers, law enforcement, and the legal system.

Ethics and dissemination: This study is approved by the University of North Carolina at Chapel Hill's Institutional Review Board (IRB) and the IRB of each participating study site. We hope to present the results of this study to the scientific community at conferences and in peer-reviewed journals.

ARTICLE SUMMARY

Strengths and limitations of this study

- To our knowledge, this is the first large-scale prospective study of experiences and recovery among women presenting for emergency care after sexual assault.
- The study obtains detailed longitudinal data from the sexual assault survivors on adverse posttraumatic neuropsychiatric sequelae (APNS) over time after sexual assault, including posttraumatic stress, pain, somatic, cognitive, and depressive symptoms.
- The study collects both biologic data to gain new insights into the pathogenesis of APNS after sexual assault, and data to develop clinical prediction tools that identify those at high risk of APNS.
- The study obtains feedback from women sexual assault survivors regarding their experience with police, the legal system, and medical providers that can be used to improve services.
- Generalizability of study findings to women sexual assault survivors who do not report/come for care after sexual assault is not known.

For peer review only

INTRODUCTION

Worldwide, an estimated 10-27% of women are sexually assaulted during their lifetime.[1] In the United States (US), as many as 1.9 million women are sexually assaulted each year.[2,3] Survivors of sexual assault experience a heavy burden of adverse posttraumatic neuropsychiatric sequelae (APNS), including fear, anxiety, and posttraumatic stress.[4] In addition, as many as half of sexual assault survivors experience clinically significant depression,[5] one quarter report suicidal ideation, and 2-19% attempt suicide.[4] Preliminary evidence from small prospective cohorts also suggests that acute and chronic pain and somatic symptoms frequently develop after sexual assault.[6-8] Amidst this tremendous physical and mental health suffering, 13-49% of sexual assault survivors develop an alcohol use disorder and 28-61% develop addiction to other substances.[9,10]

In the US, emergency care for women sexual assault survivors is most commonly provided by a nurse with specific training and expertise, termed a Sexual Assault Nurse Examiner (SANE). The emergency care of sexual assault survivors provided by a SANE includes a thorough medical and forensic history, a detailed physical examination to document evidence of trauma, evidence collection, and risk stratification and preventive interventions to reduce pregnancy and/or sexually transmitted disease. Unfortunately, no risk stratification and preventive interventions are employed to reduce APNS. This is because risk stratification tools for APNS do not exist, and because the developmental biology of ANPS is poorly understood.

This article describes the study protocol for the first large-scale emergency care-based longitudinal cohort study of recovery after sexual assault: the WHS. The goals of the study are to help address the above barriers to intervention, gain a better understanding of survivor experiences with treatment providers, law enforcement, and legal and health care systems to inform improvement, and to determine the incidence and recovery trajectories of a range of APNS.

For peer review only

METHODS/DESIGN

Study Sites

The WHS is a prospective, multi-site observational study of women presenting for emergency SANE care following sexual assault. Women are recruited at emergency care centers across the United States that are part of the Better Tomorrow Network, including: Albuquerque SANE Collaborative, Albuquerque, New Mexico; Tulsa Forensic Nurse Examiners, Tulsa, Oklahoma; Christiana Care SANE Program, Newark, Delaware; Austin SAFE, Austin, Texas; Hennepin HART, Minneapolis, Minnesota; Crisis Center of Birmingham, Birmingham, Alabama; Philadelphia SARC/Einstein Healthcare, Philadelphia, Pennsylvania; Denver Health SANE, Denver, Colorado; Wayne County SAFE, Detroit, Michigan; University of Louisville SAFE, Louisville, Kentucky; Memorial Health SANE, Colorado Springs, Colorado; DC SANE, Washington, DC; and Cone Health, Greensboro, North Carolina. Each site's IRB approved the study protocol. The Better Tomorrow Network data coordinating center is located at the University of North Carolina (UNC) at Chapel Hill North Carolina, USA, and all procedures were approved by the UNC IRB (13 – 3193).

Patient Inclusion Criteria

Women at least 18 years of age who present to a participating site for emergency care within 72 hours of assault are assessed for potential eligibility. Exclusion criteria include: inability to provide informed consent (e.g., due to intoxication, injury), pregnancy, living with one's assailant, presence of fracture, injury requiring hospital admission, inability to speak or read English, no telephone access, no mailing address, unwilling to provide a blood sample, incarceration, and other situations resulting in inability to follow the study protocols. Enrollment began in June 2015, study follow-ups are scheduled to be completed in 2020, with a target enrollment of 700 women.

Patient Screening and Initial Assessment

Treatment providers at study network sites notify on-call research associates (RAs) when a potentially eligible individual presents for emergency care after sexual assault. RAs approach potential participants at a time determined by the sexual assault survivor care provider(s). RAs provide a brief description of the study and request written consent to (1) perform a brief survey, (2) contact the survivor by telephone in 48-72 hours to explain the full study and ask if the individual is interested in participating, (3) access medical record/forensic examination information related to the sexual assault, and (4) collect blood samples (discarded if the participant does not subsequently consent to the full study at one week). Individuals are specifically informed that they are not being asked to decide about full study participation at the time of their emergency care. Participants are compensated $20 for completing the initial assessment.

RAs communicate with potential participants regarding consent for the initial evaluation either in-person or via the use of teleconsent. To obtain teleconsent, the RA introduces her/himself to the participant via live two-way video communication over tablet or laptop, and describes her/his role as a research associate in an ongoing study to learn more about the recovery process after trauma. Just as in-person, if the potential participant is willing, the RA reviews the study consent and requirements for participation and uses the online system to screen the potential participant for study eligibility. If eligible, the RA opens a copy of the initial consent on the tablet or laptop, and obtains an e-signature. The teleconsent platform complies with Health Insurance Portability and Accountability Act (HIPAA) requirements by (1) encrypting all transmitted data, (2) not storing patient information, and (3) keeping an audit trail of the consent process.[11] This teleconsent is used as a consent option because it has been shown to be a safe and valid method of obtaining consent in medical settings[11-18] and because it addresses barriers that prevent critically needed research in settings where it is difficult to consistently provide trained research staff. Teleconsent also addresses inequities created by the fact that research staff are more difficult to hire/provide in socioeconomically disadvantaged and/or rural areas.

Assessment at One Week

For peer review only

Participants who are reached by phone and express interest in study participation are scheduled for a one-week in-person assessment. Assessments take place either at the initial emergency care site or at a public community location acceptable to both the RA and study participant. At the beginning of the study visit, the RA obtains written informed consent to participate in follow-up evaluations one week, six weeks, six months, and one year after the assault and consent to utilize the blood samples obtained at the time of emergency care. After written informed consent is obtained, the one week evaluation is typically performed via a web-based self-report computer survey completed on a laptop computer. Use of a computerized self-report survey standardizes the assessment experience across RAs and sites, increases reporting of experiences that may be considered stigmatizing,[19] and facilitates honest feedback regarding the experience of being in the study. Computer self-report surveys have been shown to be a valid and acceptable means of obtaining research assessment data, including in socioeconomically disadvantaged populations recruited in emergency care settings.[20-22] The beginning of each web-based assessment includes introductory practice questions using the different questionnaire formats. After the completion of these practice questions, the RA moves to another location in the room, so that the study participant has privacy when recording her computer questionnaire responses. Participants are compensated $50 for completing the one-week assessment.

Assessment at Six Weeks, Six Months, and Twelve Months

Follow-up survey assessments at six weeks, six months, and twelve months may be completed in-person, or if the participant prefers, via logging into a secure website on a computer of their choice, or over the phone. The format of the web-based computer self-report surveys completed online are identical to the web-based computer self-report surveys completed by participants who elect to complete surveys in-person. Each online assessment contains specific completion instructions. Participants who complete follow-up assessments online independently may call the toll-free study phone number if they have any questions while completing the assessment. Participants are encouraged to complete these follow-up assessments either in-person or online, to maximize standardization. However, if participants are unable to complete these follow-up assessments either in-person or online, they may also complete them via telephone interview. Participants are compensated $50 for completing each follow-up assessment.

Contact Information Updates at Three Months and Nine Months

Participants are contacted three months and nine months after enrollment, and are asked to update their contact information. Participants are compensated $10 for each follow-up request.

Confidentiality and Security of Participant Data

All paper forms are stored in locked file cabinets with specially-made keys at the Better Tomorrow Network Data Coordinating Center in Chapel Hill, NC. These forms are labeled with participant ID only. Consent forms are stored separately, because they contain personally identifying information. Study data are housed on a secure, firewalled server dedicated for research use only and maintained by the University of North Carolina School of Medicine. Data are stored on an SQL Server, with encryption and full daily and nightly backups. Both the Application server and the Database server are located behind The University of North Carolina School of Medicine firewall, and only approved site users have access via ASP.NET Forms Authentication or Windows Integrated Authentication.

Patient and Public Involvement

The study protocol was designed in collaboration with sexual assault nurse examiners; this team included sexual assault survivors. The study was reviewed and funded by the National Institutes of Health, which considers public funding priorities and maximizing the public good in scientific review and funding decisions. As described below, study participants provide feedback on burden and experiences in the research study at the end of each assessment. Study results are disseminated to the public via presentations, publications, and media coverage, and via provision of study results to individual sexual assault treatment facilities and to state coalitions against sexual assault in states in which participating care sites are located.

For peer review only

Study Assessments

Assessments and data collection at the time of emergency care

Pain severity (0-10 numeric rating scale score; Table 1) is assessed in each of 23 body regions using an adapted version of the Regional Pain Scale (RPS).[23] This scale has been shown to be a valid, reliable measure of pain location and distribution.[23] Pain severity in the head and face, breast area, pelvic/genital region, hands, and feet are also assessed. Blood DNA and RNA are collected using PAXgene DNA (8.5cc) and RNA (2.5cc) storage tubes, respectively. Samples are frozen at the study site and are shipped in batches to the University of North Carolina. A barcode-based study tracking system tracks each tube throughout the storage and shipment process to maintain chain of custody.

Medical records regarding emergency care after sexual assault are password protected and then uploaded to the encrypted, secure study database by the site RA. If some of the participant's emergency care was provided at a treatment location separate from the location of the SANE exam, these records are also obtained by study staff. Medical record data are abstracted by RAs using detailed protocols and variable definitions. Data extracted include detailed information regarding the assault, medical care received, past medical history, and assailant relationship with the survivor (e.g., relatives, current or former romantic partners, friend/acquaintance, planned first encounter, stranger, or unknown.) Quality assurance and control of data abstraction are assessed via double data entry and discrepancies are adjudicated by the study PI.

Assessments at One Week, Six Weeks, Six Months, and One Year

Assessments at one week, six weeks, six months, and one year include the following:

General physical and mental health status: The Patient-Reported Outcomes Measurement Information System (PROMIS) Global Health assessment is used to assess general pre-assault health at the one-week follow-up. At subsequent time points, this measure is used to assess health status.[24]

Somatic symptoms: Pre-injury somatic symptoms during the week prior to the sexual assault are assessed at one-week follow-up using a 21-question symptom inventory. Somatic symptoms during the past week are additionally assessed at each follow-up time point.

Posttraumatic stress disorder symptoms, past trauma, and resilience: Lifetime sexual assault and other trauma exposure and PTSD history are assessed at one week using questions from an adapted version of the Life Events Checklist.[25] Lifetime PTSD symptoms related to prior trauma are assessed with an abbreviated and adapted version of the PCL-5[26,27] that explicitly instructs the respondent to focus only on symptoms related to prior stressors, not the recent sexual assault. An adapted version of the Trait Resilience scale of Kessler, et al [28-30] is administered at one week and the Adverse Childhood Experiences (ACE [31,32]) Score Calculator is administered at six weeks to assess whether women experienced childhood sexual assault or other forms of trauma.[32] Recent sexual assault-related PTSD symptoms are assessed using the PCL-5[27,33] with the addition of the two DSM-IV-related items from the PCL-C[26,27,34].

Depressive and other anxiety-related assessments symptoms: Short-form versions of the PROMIS Depression and Anxiety assessments[24] (Short Form 8b and Short Form 8a, respectively) are used at the one-week follow-up to assess depressive and anxiety symptom burden in the week prior to assault. These assessments are also used at the six-week, six-month, and twelve-month follow-ups to assess symptoms during the past week. In addition, a brief version of the Anxiety Sensitivity Index - 3 (ASI-3) is administered at one week follow-up.[35,36]

Pain symptoms: The same assessment method used to evaluate the severity and location of pain at the time of SANE exam are used to evaluate both reported pre-assault pain and post-assault pain symptoms. Pain symptoms assessed at each time point are specifically termed "pain/tenderness" and "pain or aching", as past experience with sexual assault survivors indicates that some survivors otherwise interpret questions about "pain" to include "emotional pain." At the one-week assessment, after being asked about the location and severity of any pain symptoms since the assault, participants are also asked about the location and severity of any pain symptoms during the week before assault. If participants report pain in a body region at six-week, six-month, or twelve-month follow-up, after rating the severity of pain in that region participants are asked to indicate if this pain is related to the sexual assault. In addition to pain severity assessments in each body region, at each follow-up

For peer review only

timepoint overall pain is also assessed using a 0-10 NRS and pain interference during the past week is assessed using questions from the Brief Pain Inventory.[37]

Substance use: Drug, alcohol, and tobacco use during the month prior to the month of assault are assessed at the one-week follow-up using an adapted version of the CIDI-SC assessment of substance use disorder of Kessler, et al.[29,30] This time point was chosen to exclude the timeframe of the assault. At all subsequent follow-ups, use during the past month is assessed.

Demographic information: Information including age, race/ethnicity, education level, income level, marital status, work status, and height and weight are obtained from questionnaire items and the medical record.

Past medical history: Participant past medical history is obtained from the medical records.

Medication use: Participant medication use is assessed at each follow-up evaluation.

Experiences of sexual assault survivors with care providers and the health care and legal systems: Sexual assault survivor experiences, including experiences with the SANE program, legal system, and police, as well as health services utilization and injuries since assault, are assessed using standardized questionnaires. Legal system assessments include an evaluation of experiences with reporting the assault to police, interactions with prosecutors, whether there was an assault investigation was performed (and if not performed, reason(s) the survivor was told there was no investigation), and status/outcomes of the legal process.

Sexual assault survivor experiences with the study in general and with individual follow-up assessments: After each interview, participants complete an adapted version of the Reactions to Research Participation Questionnaire (RRPQ)[38,39] and open-ended questions about their experiences as a study participant and sexual assault survivor. Open-ended questions ask survivors the following: "Is there anything about this survey that we could do better?" and, "What do you think is most important for researchers to understand about your experience since the assault?"

Analyses

Study results will be shared with the scientific community via conferences and peer-reviewed journal articles. Planned analyses fall into four broad areas.

1. Assess the incidence over time of a range of APNS, using both traditional and experimental/state-of-the-art classification systems. No large-scale emergency care-based multi-site studies of sexual assault survivors have been conducted, thus the incidence of the full spectrum of APNS over time after sexual assault remains poorly understood. This study will assess a range of APNS, as well as multivariate patterns of comorbidity among these outcomes and the impact of these outcomes on general physical and mental health over time. APNS will be assessed using both traditional classifications (e.g., posttraumatic stress disorder, depression, pain, and post-concussion symptoms), and also more discrete, homogenous outcomes (e.g., avoidance, re-experiencing, numbing, hopelessness, etc.). These homogenous outcomes will, to the extent possible, build upon the National Institute of Mental Health Research Domain Criteria (RDoC) classification system (https://bit.ly/2pudCZH). Subsequent analyses will attempt to group sexual assault survivors according to the combinations of these discrete outcomes as they develop. Classification systems such as RDoC attempt to overcome two important limitations to traditional classifications: (1) they are not indexed to specific biological processes or components of brain functioning, which often evolved based

2. Gain new understanding of the pathogenesis of adverse posttraumatic neuropsychiatric outcomes after sexual assault. As noted above, a major barrier to developing more effective preventive interventions is that the pathogenesis of APNS after sexual assault remains poorly understood. An evaluation of risk factors and developmental processes involved in the temporal unfolding of adverse posttraumatic neuropsychiatric outcomes after sexual assault will be evaluated using both traditional and molecular epidemiologic methods, with complementary cell culture and animal mechanistic studies where valuable. Available data indicate that biologic samples collected in the immediate aftermath of trauma can provide valuable insights into the pathogenesis of APNS (e.g.,[40-50]). The goal of studies performed with these data (e.g., genetic, epigenetic, gene expression, miRNA studies) will be to gain improved understanding to inform the development of more effective preventive/recovery interventions for sexual assault survivors.

For peer review only - http://bmjopen.bmj.com/site/about/guidelines.xhtml

3. Derive risk prediction tools that identify sexual assault survivors at high risk of specific adverse posttraumatic outcome(s). For more than 50 years, women sexual assault survivors presenting for emergency care after assault have been risk stratified for pregnancy and sexually transmitted disease, and those at high risk have received preventive interventions. However, no such risk stratification tools exist to help identify women at high risk of APNS.[4] Survivor assault characteristics, demographic characteristics, life history, pre-assault health status, acute posttraumatic symptoms, and biologic characteristics will be used in an effort to develop clinical decision support tools that identify survivors at high risk of one or more APNS. These clinical decision support tools will be developed using machine learning (ML) methods,[51] training and validation samples, and internal cross-validation of training samples to minimize over-fitting. A number of different machine learning algorithms will be examined, such as naïve Bayes, ensemble regression trees (random forests), penalized regression (elastic net), algorithms that allow for nonlinearities and non-additivities in stepwise trees with embedded splines (gradient boosting), and support vector machines. The value of combining results across these different algorithms using the super learner ensemble method will also be assessed.[52-56] Tiering and targeting will be used to try to limit the number of items needed in prediction tools. Tiering refers to nested ML analyses based on successively adding more costly predictors to the models, where cost is defined in terms of both time (e.g., number of questions that SANE needs to ask to complete) and processing (e.g., costs of genetic testing, neuroimaging, etc.). Targeting refers to determining subsets of patients that vary in the extent to which prediction accuracy over a clinical decision threshold varies depending on a given level of tiering. For example, screening tests are often used to determine whether individual patients need more complex and expensive tests. The equivalent in our context will be to determine the cross-validated predicted values based on initial predictors that indicate the need for further data.

4. Obtain feedback from sexual assault survivors regarding their experiences with health care providers, law enforcement, and the legal system, and their experiences with the present research study. The lack of large-scale research networks for sexual assault survivors has hampered the ability to obtain feedback from survivors regarding services they receive. Such feedback is critical for continued quality improvement and to ensure that health, law enforcement, and legal systems designed for sexual assault survivors are indeed "survivor-centered." Similarly, it is critical for research studies to ensure that their protocols are not viewed as negative experiences by sexual assault survivor cohorts taking part in them. Analyses will be performed which evaluate survivor experiences with these services and with the research protocol. Of note, for all analyses in which substantial missing data is present, sensitivity analyses will be performed evaluating analyses results with and without data imputation for missing values.

Ethics and Dissemination

Although the safety of participants is of paramount importance to any research study, this is a matter of particular concern in studies of traumatized individuals. Study participants are told that they may choose not to answer any questions that cause discomfort and that they will be paid the full financial incentive regardless of whether or not they decide to skip self-report questionnaire items or stop the questionnaire. As noted above, study participants who choose to participate at the time of the emergency care provide written or electronic informed consent only for the initial evaluation. At the beginning of the one-week assessment, in-person written consent is obtained to perform the one-week assessment and follow-up assessments. The research assessments never include details of/questions regarding the assault experience itself. This information is obtained from emergency care records. During enrollment in the study, participants are informed that they will be contacted to learn more about the recovery process after trauma. The study is framed in terms of "recovery," as we believe that it is very important to help create an expectation of healing in study participants since previous research suggests that participant expectation may potentially influence long-term outcomes after trauma.[57] One-week assessments are performed via telephone or self-report computer-based questionnaire. Subsequent surveys at six weeks, six months, and one year are also offered either via telephone or self-report computer-based questionnaire. This increases privacy and anonymity and encourages candid participant feedback regarding study participation. Assessment modalities (i.e.., in-person, online, telephone) are offered depending on participants' preference. This methodology balances

For peer review only

our goals of providing as much anonymity as possible while allowing those participants the opportunity to continue to with the study who have travel issues or do not have internet access at a follow-up time point.

Study participants receive the exact same survivor services and follow-up referrals/care as other survivors presenting for care. All survivors are actively referred for mental and physical health services through usual post-assault care follow-up services managed by their emergency care providers and are encouraged to utilize a rape crisis advocate to help them receive this care. Participants who report substantial distress and/or who appear to be developing significant anxiety and/or depressive symptoms are encouraged to utilize local mental health services. In addition, study participants are provided with a book (free of charge), *Recovering from Rape*[58] or *Life, Reinvented*,[59] which is written specifically to help women in the early aftermath of sexual assault.

Concluding Summary

The WHS is a unique large-scale emergency care-based longitudinal cohort study of recovery in the 12 months after sexual assault. The study seeks to increase understanding of the incidence, pathogenesis, and trajectories of adverse neuropsychiatric sequelae, to develop clinical decision support tools that effectively predict such outcomes, and to obtain feedback from survivors regarding the services they receive. Study success is based on our study participants, who share their experiences with the understanding that researchers will use this information to try to improve the care of survivors in the future. We are grateful for their efforts and recognize our responsibility.

For peer review only

FUNDING

This work was supported by NIH R01 AR064700. The content is solely the responsibility of the authors and does not necessarily represent the official views of the NIH.

COMPETING INTERESTS

In the past 3 years, Dr. Kessler received support for his epidemiological studies from Sanofi Aventis; was a consultant for Johnson & Johnson Wellness and Prevention, Sage Pharmaceuticals, Shire, Takeda; and served on an advisory board for the Johnson & Johnson Services Inc. Lake Nona Life Project. Kessler is a co-owner of DataStat, Inc., a market research firm that carries out healthcare research.

CONTRIBUTORS

Samuel McLean conceived of the study. Nicole Short, Jenyth Sullivan, April Soward, Kenneth Bollen, Israel Liberzon, Sandra L. Martin, Sheila A.M. Rauch, Kathy Bell, Catherine Rossi, Megan Lechner, Ronald C. Kessler, Carissa Novak, Kristen Witkemper, and Samuel McLean helped to design the final study protocol. All authors reviewed the manuscript.

For peer review only

Table 1

Study question domains and self-report measures administered at each time point

Domain	Measure	Initial	1-Wk	6-Wk	6-Mo	12-Mo
Assault Characteristics	Data extraction from medical record	X				
Acute Pain	Pain Severity Numeric Rating Scale[60]	X	X	X	X	X
Pain Related to the Assault	Pain Severity Numeric Rating Scale[60]		X	X	X	X
Pain Interference	Brief Pain Inventory[61]		X	X	X	X
Pre-Assault Pain	Pain Severity Numeric Rating Scale[60]		X			
Current Medication Use	Standard Items		X	X	X	X
Survivor Experience	SANE Care, advocate, post-exposure prophylaxis		X			
	Experience with police, post-exposure prophylaxis, health-services utilization, out-of-pocket expenses			X		
	Experience with police, health services utilization, out-of-pocket expenses				X	
	Experience with police, experience with legal system, health services utilization, out-of-pocket expenses					X
Somatic Symptoms	Numeric rating scale score of 21 common symptoms		X	X	X	X
Pre-Assault Somatic Symptoms	Standard Items		X			
Posttraumatic Stress Symptoms	PTSD Checklist – Situation (PCL-S)[26,27]		X	X	X	X
Pre-Assault Trauma Exposure	Adapted version of the Life Events Checklist[25]		X			
Pre-Assault Posttraumatic Stress	Adapted version of PCL-5[26,27]		X			
Childhood Trauma Exposure	Childhood Adversity Exposures (ACE)[31]			X		
Resilience	Trait Resiliency[28]		X			
General Health	PROMIS Global Health – Physical Component[24]		X	X	X	X
New Health Status	Standard items			X	X	X

For peer review only

Depression	PROMIS – Short Form 8b[24]	X	X	X	X
Anxiety	PROMIS – Short Form 8a[24]	X	X	X	X
Substance Use	Adapted version of Composite International Diagnostic Interview Screening Scales (CIDI-SC)[29]	X	X	X	X
Demographics	Standard items	X			
Research Experience	Reactions to Research Participation Questionnaire Revised[39]	X	X	X	X

Note: SANE = sexual assault nurse examiner; PTSD = posttraumatic stress disorder; PROMIS = Patient-reported Outcomes Measurement Information System; CIDI-SC = Composite International Diagnostic Interview – Screening Scale.

For peer review only

REFERENCES

1. García-Moreno C, World Health Organization. Dept. of Gender and Women's Health. WHO multi-country study on women's health and domestic violence against women : initial results on prevalence, health outcomes and women's responses. Geneva: World Health Organization; 2005.
2. Breiding MJ, Smith SG, Basile KC, Walters ML, Chen J, Merrick MT. Prevalence and characteristics of sexual violence, stalking, and intimate partner violence victimization—National Intimate Partner and Sexual Violence Survey, United States, 2011. American journal of public health 2015;105:E11.
3. Peterson C, DeGue S, Florence C, Lokey CN. Lifetime economic burden of rape among US adults. American journal of preventive medicine 2017;52:691-701.
4. Campbell R, Dworkin E, Cabral G. An ecological model of the impact of sexual assault on women's mental health. Trauma, Violence, & Abuse 2009;10:225-46.
5. Acierno R, Brady K, Gray M, Kilpatrick DG, Resnick H, Best CL. Psychopathology following interpersonal violence: A comparison of risk factors in older and younger adults. Journal of Clinical Geropsychology 2002;8:13-23.
6. Ballina LE, Ulirsch JC, Soward AC, et al. mu-Opioid Receptor Gene A118G Polymorphism Predicts Pain Recovery After Sexual Assault. J Pain 2012.
7. McLean SA, Soward AC, Ballina LE, et al. Acute severe pain is a common consequence of sexual assault. J Pain 2012;13:736-41.
8. Ulirsch JC, Ballina LE, Soward AC, et al. Pain and somatic symptoms are sequelae of sexual assault: results of a prospective longitudinal study. Eur J Pain 2014;18:559-66.
9. Ullman SE, Filipas HH, Townsend SM, Starzynski LL. Psychosocial correlates of PTSD symptom severity in sexual assault survivors. Journal of traumatic stress 2007;20:821-31.
10. Ullman SE, Filipas HH, Townsend SM, Starzynski LL. Correlates of comorbid PTSD and drinking problems among sexual assault survivors. Addictive behaviors 2006;31:128-32.
11. Welch B, Marshall E, Qanungo S, et al. Teleconsent: A novel approach to obtain informed consent for research. Contemporary Clinical Trials Communications 2016;3:74-9.
12. Schenker Y, Meisel A. Informed consent in clinical care: practical considerations in the effort to achieve ethical goals. JAMA 2011;305:1130-1.
13. Bobb MR, Van Heukelom PG, Faine BA, et al. Telemedicine provides noninferior research informed consent for remote study enrollment: a randomized controlled trial. Academic Emergency Medicine 2016;23:759-65.
14. Sonne SC, Andrews JO, Gentilin SM, et al. Development and pilot testing of a video-assisted informed consent process. Contemporary clinical trials 2013;36:25-31.
15. Rojas SV, Gagnon M-P. A systematic review of the key indicators for assessing telehomecare cost-effectiveness. Telemedicine and e-Health 2008;14:896-904.
16. Witherspoon E. UNC's CTSA hosts experts in telemedicine and health information technology. News Room-UNC Healthcare Vital Signs 2010.

For peer review only

17. Cone Health. Health Online: How the Internet is Revolutionizing Patient Service. 2017.
18. Narum E. Making the Grade: School-Based Telemedicine and Parental Consent. Making the Grade. San Diego Law Review 2016;53:745-51.
19. Turner CF, Ku L, Rogers SM, Lindberg LD, Pleck JH, Sonenstein FL. Adolescent sexual behavior, drug use, and violence: increased reporting with computer survey technology. Science (New York, NY) 1998;280:867-73.
20. Gregor MA, Shope JT, Blow FC, Maio RF, Weber JE, Nypaver MM. Feasibility of using an interactive laptop program in the emergency department to prevent alcohol misuse among adolescents. Annals of Emergency Medicine 2003;42:276-84.
21. McLean SA, Blow FC, Walton MA, et al. Rates of At‐risk Drinking among Patients Presenting to the Emergency Department with Occupational and Nonoccupational Injury. Academic Emergency Medicine 2003;10:1354-61.
22. Rhodes KV, Lauderdale DS, Stocking CB, Howes DS, Roizen MF, Levinson W. Better health while you wait: a controlled trial of a computer-based intervention for screening and health promotion in the emergency department. Annals of Emergency Medicine 2001;37:284-91.
23. Wolfe F. Pain extent and diagnosis: development and validation of the regional pain scale in 12,799 patients with rheumatic disease. The Journal of rheumatology 2003;30:369-78.
24. Cella D, Riley W, Stone A, et al. The Patient-Reported Outcomes Measurement Information System (PROMIS) developed and tested its first wave of adult self-reported health outcome item banks: 2005–2008. Journal of clinical epidemiology 2010;63:1179-94.
25. Gray MJ, Litz BT, Hsu JL, Lombardo TW. Psychometric properties of the life events checklist. Assessment 2004;11:330-41.
26. Weathers F, Litz B, Herman D, Huska J, Keane T. The PTSD checklist (PCL): Reliability, validity, and diagnostic utility. Annual Convention of the International Society for Traumatic Stress Studies. San Antonio, TX1993.
27. Weathers FW, Litz BT, Keane TM, Palmieri PA, Marx BP, Schnurr PP. The PTSD checklist for DSM-5 (PCL-5). National Center for PTSD; 2013.
28. Campbell‐Sills L, Kessler RC, Ursano RJ, et al. Predictive validity and correlates of self‐assessed resilience among US Army soldiers. Depression and anxiety 2018;35:122-31.
29. Kessler RC, Santiago PN, Colpe LJ, et al. Clinical reappraisal of the Composite International Diagnostic Interview Screening Scales (CIDI‐SC) in the Army Study to Assess Risk and Resilience in Servicemembers (Army STARRS). International journal of methods in psychiatric research 2013;22:303-21.
30. Kessler RC, Calabrese JR, Farley P, et al. Composite International Diagnostic Interview screening scales for DSM-IV anxiety and mood disorders: Cambridge Univ Press; 2013.
31. Felitti VJ, Anda RF, Nordenberg D, et al. Relationship of childhood abuse and household dysfunction to many of the leading causes of death in adults: The Adverse Childhood Experiences (ACE) Study. American journal of preventive medicine 1998;14:245-58.

For peer review only

32. Butchart A, Harvey AP, Mian M, Furniss T. Preventing child maltreatment: a guide to taking action and generating evidence. 2006.
33. Blanchard EB, Jones-Alexander J, Buckley TC, Forneris CA. Psychometric properties of the PTSD Checklist (PCL). Behaviour research and therapy 1996;34:669-73.
34. Weathers FW, Huska JA, Keane TM. PCL-C for DSM-IV. Boston: National Center for PTSD-Behavioral Science Division 1991.
35. Reiss S, Peterson RA, Gursky DM, McNally RJ. Anxiety sensitivity, anxiety frequency and the prediction of fearfulness. Behavior Research and Therapy 1986;24:1-8.
36. Taylor S, Zvolensky MJ, Cox BJ, et al. Robust dimensions of anxiety sensitivity: development and initial validation of the Anxiety Sensitivity Index-3. Psychol Assess 2007;19:176-88.
37. McCaffrey M, Pasero C. Pain: Clinical Manual. St. Louis, MO: Mosby, Inc.; 1999.
38. Griffin M, Resick P, Waldrop A, Mechanic M. Participation in trauma research: is there evidence of harm? J Trauma Stress 2003;16:221.
39. Newman E, Kaloupek D. Reactions to research participation questionnaire. Available from E Newman, Department of Psychology, University of Tulsa 1996;600:74114-3189.
40. Linnstaedt SD, Walker MG, Parker JS, et al. microRNA circulating in the early aftermath of motor vehicle collision predict persistent pain development and suggest a role for microRNA in sex-specific pain differences. Molecular Pain 2015;In Press.
41. Linnstaedt SD, Riker KD, Rueckeis CA, et al. A Functional riboSNitch in the 3' Untranslated Region of FKBP5 Alters MicroRNA-320a Binding Efficiency and Mediates Vulnerability to Chronic Post-Traumatic Pain. J Neurosci 2018;38:8407-20.
42. Linnstaedt SD, Hu J, Bortsov AV, et al. mu-Opioid Receptor Gene A118 G Variants and Persistent Pain Symptoms Among Men and Women Experiencing Motor Vehicle Collision. J Pain 2015;16:637-44.
43. Linnstaedt SD, Bortsov AV, Soward AC, et al. CRHBP polymorphisms predict chronic pain development following motor vehicle collision. Pain 2016;157:273-9.
44. Ballina LE, Ulirsch JC, Soward AC, et al. mu-Opioid receptor gene A118G polymorphism predicts pain recovery after sexual assault. J Pain 2013;14:165-71.
45. Ulirsch JC, Weaver MA, Bortsov AV, et al. No man is an island: living in a disadvantaged neighborhood influences chronic pain development after motor vehicle collision. Pain 2014;155:2116-23.
46. Orrey DC, Bortsov AV, Hoskins JM, et al. Catechol-O-methyltransferase genotype predicts pain severity in hospitalized burn patients. J Burn Care Res 2012;33:518-23.
47. Bortsov AV, Smith JE, Diatchenko L, et al. Polymorphisms in the glucocorticoid receptor co-chaperone FKBP5 predict persistent musculoskeletal pain after traumatic stress exposure. Pain 2013;154:1419-26.
48. Bortsov AV, Diatchenko L, McLean SA. Complex multilocus effects of catechol-O-methyltransferase haplotypes predict pain and pain interference 6 weeks after motor vehicle collision. Neuromolecular Med 2014;16:83-93.

For peer review only

49. Bortsov A, Diatchenko L, Swor RA, et al. COMT haplotypes predict pain intensity and interference 6 weeks after motor vehicle collision. Annual Meeting of the American Pain Society. New Orleans, LA2013.
50. Auvergne L, Bortsov AV, Ulirsch JC, et al. Association of Epidemiologic Factors and Genetic Variants Influencing Hypothalamic-Pituitary-Adrenocortical Axis Function With Postconcussive Symptoms After Minor Motor Vehicle Collision. Psychosom Med 2016;78:68-78.
51. Hastie T, Tibshirani R, Friedman JH. The elements of statistical learning : data mining, inference, and prediction. New York: Springer; 2009.
52. Kessler RC, Rose S, Koenen KC. How well can post‐traumatic stress disorder be predicted from pre‐trauma risk factors? An exploratory study in the WHO. World Mental Health Surveys World Psychiatry;2014:265‐74.
53. Kessler RC, Warner CH, Ivany C. Predicting suicides after psychiatric hospitalization in US Army soldiers: the army study to assess risk and resilience in service members (Army STARRS). JAMA psychiatry;2015:49–57.
54. van der Laan MJ PE, Hubbard AE. Super learner. Stat Appl Genet Mol Biol 2007:25.
55. Kessler R, van Loo H, Wardenaar K. Testing a machine-learning algorithm to predict the persistence and severity of major depressive disorder from baseline self-reports. Molecular Psychiatry in press.
56. Koenen KC, Kessler RC, Shalev AY. RO1:Identifying risk factors for ptsd by pooled analysis of current prospective studies. NIH:2014–9.
57. Mittenberg W, DiGiulio D, Perrin S, Bass A. Symptoms following mild head injury: expectation as aetiology. J Neurol Neurosur Ps 1992;55:200.
58. Ledray LE. Recovering from rape: Macmillan; 1994.
59. E. C. Life, Reinvented: Quantum Publishing Group; 2014.
60. Bijur PE, Latimer CT, Gallagher EJ. Validation of a verbally administered numerical rating scale of acute pain for use in the emergency department. Academic Emergency Medicine 2003;10:390-2.
61. Keller S, Bann CM, Dodd SL, Schein J, Mendoza TR, Cleeland CS. Validity of the brief pain inventory for use in documenting the outcomes of patients with noncancer pain. The Clinical journal of pain 2004;20:309-18.

For peer review only
